# Point-of-care ultrasound defines gastric content in elective surgical patients with type 2 diabetes mellitus: a prospective cohort study

**DOI:** 10.1186/s12871-019-0848-x

**Published:** 2019-10-10

**Authors:** Li Zhou, Yi Yang, Lei Yang, Wei Cao, Heng Jing, Yan Xu, Xiaojuan Jiang, Danfeng Xu, Qianhui Xiao, Chunling Jiang, Lulong Bo

**Affiliations:** 1 Department of Anesthesiology and Translational Neuroscience Center, West China Hospital, Sichuan University, Chengdu, 610041 Sichuan China; 2 Department of Anesthesiology, Cheng Du Shang Jin Nan Fu Hospital, Chengdu, 610000 Sichuan China; 3 Faculty of Anaesthesiology, Changhai Hospital, Naval Medical University, Shanghai, 200433 China

**Keywords:** Type 2 diabetes mellitus, Gastric emptying, Regurgitation and aspiration, Ultrasonography

## Abstract

**Background:**

Delayed gastric emptying and the resultant “full stomach” is the most important risk factor for perioperative pulmonary aspiration. Using point-of-care gastric sonography, we aimed to investigate the prevalence of full stomach and its risk factors in elective surgical patients with type 2 diabetes.

**Methods:**

Type 2 diabetic and non-diabetic elective surgical patients were included from July 2017 to April 2018 in a 1:1 ratio. The study was retrospectively registered at July 2017, after enrollment of the first participant. Gastric ultrasound was performed 2 h after ingesting clear fluid or 6 h after a light meal. Full stomach was defined by the presence of gastric content in both semi-recumbent and right lateral decubitus positions. For patients with full or intermediate stomach, consecutive ultrasound scan was performed until empty stomach was detected. Logistic regression analyses were used to identify risk factors associated with full stomach.

**Results:**

Fifty-two type 2 diabetic and fifty non-diabetic patients were analyzed. The prevalence of full stomach was 48.1% (25/52) in diabetic patients, with 44.0% for 2-h fast after clear fluid and 51.9% for 6-h fast after a light meal, significantly higher than 8% (4/50) in non-diabetic patients (*P* = 0.000). The average time to empty stomach in diabetic patients was 146.50 ± 40.91 mins for clear liquid and 426.50 ± 45.25 mins for light meal, respectively. Further analysis indicated that presence of diabetes-related eye disease was an independent risk factor of full stomach in diabetic patients (OR = 4.83, *P* = 0.010).

**Conclusions:**

Almost half of type 2 diabetic patients have a full stomach following the current preoperative fasting guideline. Preoperative ultrasound assessment of gastric content in type 2 diabetic patients is suggested, especially for those with diabetes -related eye disease.

**Trial registration:**

The trial was registered at www.clinicaltrials.gov with registration number NCT03217630. Retrospectively registered on 14th July 2017.

## Introduction

Gastric emptying is known to be delayed in patients with diabetes mellitus [1, 2]. Approximately 30–50% of patients with longstanding diabetes mellitus have significantly prolonged gastric emptying time, as measured by radioisotope examination [3, 4]. Delayed gastric emptying and the resultant “full stomach” is the most important risk factor for perioperative regurgitation and aspiration, which remains a common, disastrous complication with high morbidity and mortality in patients undergoing general anesthesia. Consequently, American Society of Anesthesiologists (ASA) released preoperative fasting guidelines for healthy patients undergoing elective surgery [5], in order to reduce gastric content volume and minimize the risk of aspiration. However, there are still many situations where the ASA fasting guidelines may be not suitable, including urgent or emergency situations and medical conditions, e.g., diabetes mellitus, which is associated with delayed gastric emptying.

Recent studies have shown that ultrasound examination can be used for the accurate assessment of gastric volume and content with high intra- and inter-rater reliability in healthy subjects [6–8], surgical patients [9], and others [10, 11]. As a novel point-of-care application, ultrasound sonography allows anesthesiologists to evaluate a patient’s gastric content and volume at the bedside, and helps guide anesthetic and airway management [12–14]. We hypothesize that ultrasound sonography will be helpful to determine the gastric content in elective surgical patients with type 2 diabetes mellitus.

Using this non-invasive technique for the assessment of gastric content, we aimed to determine the prevalence of full stomach following the present fasting guidelines in elective adult surgical patients with type 2 diabetes mellitus, and to investigate associated risk factors for delayed gastric emptying, in this prospective cohort study.

## Materials and methods

After obtaining Ethics approval (Ethical Committee N° 2017–141) from the Ethical Committee of West China Hospital, Chengdu, China (Chairperson Prof MZ. Liang) on 16 June 2017, we conducted this prospective cohort study in West China hospital according to the principles expressed in the Declaration of Helsinki from July 2017 to April 2018. The study was retrospectively registered at July 2017, after enrollment of the first participant. Type 2 diabetic and non-diabetic patients admitted to the surgical department were screened and recruited to participate in the study. Inclusion criteria were as follows: type 2 diabetic (two fasting plasma glucose concentration ≥ 7 mmol/L or casual plasma glucose concentration ≥ 11.1 mmol/L with classic symptoms of hyperglycemia) [15] or non-diabetic patient; age ≥ 18 yr; ASA physical status I-III; body mass index (BMI) < 35 Kg/m^2^; elective surgery; be able to understand the rationale of the study and provide informed consent. Exclusion criteria were as follows: pregnancy; a history of upper gastrointestinal disease or previous surgery on the esophagus, stomach or upper abdomen; documented abnormalities of the upper gastrointestinal tract such as gastric tumors; recent upper gastrointestinal bleeding (within the preceding 1 month); taking medicines that may delay gastric emptying (e.g., anticholinergic agents, opioid); hypothyroidism. Written informed consent was obtained from all included subjects.

Eligible type 2 diabetic and non-diabetic subjects were recruited in a 1:1 ratio. Subjects in both groups were fasted overnight (at least 10 h) from the last meal. After enrollment, patients will be randomized to ingesting either clear fluid or light meal (a standardized portion of noodles or toast, and clear fluid). Randomization was performed using computer-generated random numbers and group assignments were delivered in sealed, opaque envelopes. An attending anesthesiologist, who had an experience with at least 100 gastric ultrasound examinations previously, performed all ultrasound examinations in the study. The anesthesiologist was blinded to group allocation or the history of the participants. Ultrasound examinations were carried out 2 h after ingesting clear fluid or 6 h after a light meal, according to preoperative fasting guidelines by ASA released in early 2017 [5].

### Ultrasound examination

**Fig. 1:**
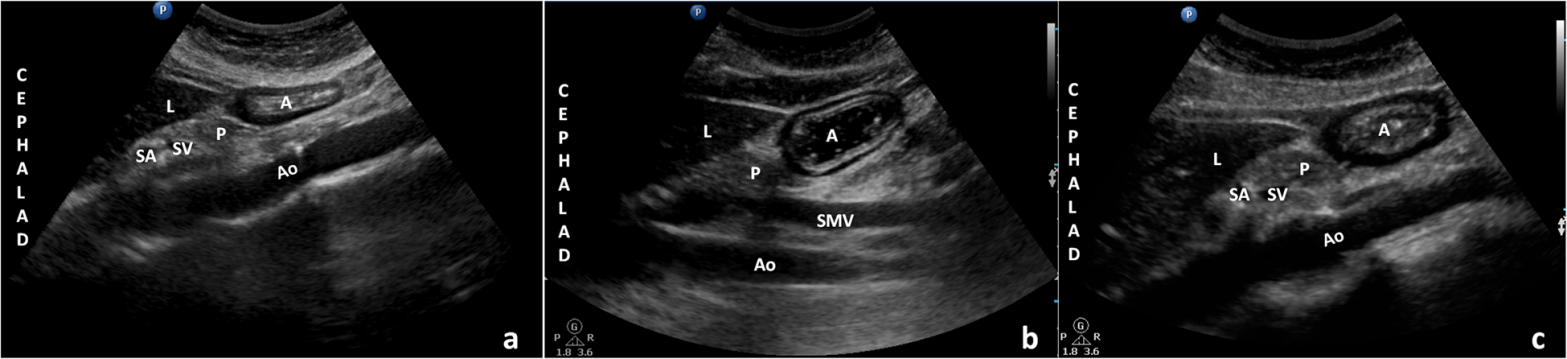
Sonographic image of the gastric antrum in the semi-recumbent position. Ultrasound examinations were conducted with a low-frequency curvilinear array probe from a Philips (CX50), showing an empty gastric antrum (**a**), liquid (**b**) and semi-solid (**c**) food in the gastric antrum. A sagittal cross-section of the antrum in a plane, including the left lobe of the liver anteriorly, the pancreas and aorta posteriorly was acquired. A, antrum; L, liver; P, pancreas; SA, splenic artery; SV, splenic vein; Ao, aorta**;** SMV, superior mesenteric vein

Ultrasound examinations were conducted with a low-frequency (2-5 MHz) curvilinear array probe from a Philips (CX50) (Bothell, WA, USA). As previously described [16], a sagittal cross-section of the antrum in a plane including the left lobe of the liver anteriorly, and the pancreas and aorta posteriorly was acquired. All quantitative and qualitative examinations were performed in the semi-recumbent and then the right lateral decubitus (RLD) positions. A three-point grading scale described by Perlas was used for the qualitative assessment: Grade 0, no gastric content was detected in antrum in either semi-recumbent or RLD position (Fig. 1a); Grade1, the gastric content was detected in the RLD only; Grade2, the content was detected in both semi-recumbent and RLD positions (Fig. 1b – Fig. 1c) [17]. For the quantitative assessment, the antral cross-sectional area (CSA) was calculated as follows [18]: measuring the anterior-posterior (D1) and cranio-caudal diameters (D2) of the antrum at antral resting, from serosa to serosa, using the formula: Antral cross-sectional area = D1 × D2× π/4.

Ultrasound examinations were conducted with a low-frequency curvilinear array probe from a Philips (CX50) (Fig. 1), showing an empty gastric antrum (a), liquid (b) and semi-solid(c) food in the gastric antrum. A sagittal cross-section of the antrum in a plane, including the left lobe of the liver anteriorly, the pancreas and aorta posteriorly was acquired. A, antrum; L, liver; P, pancreas; SA, splenic artery; SV, splenic vein; Ao, aorta; SMV, superior mesenteric vein.

The stomach was considered as empty in either Perlas Grade 0 regardless of the CSA, or Grade 1 with CSA < 340 mm^2^. Intermediate stomach contents were defined as Grade 1 with CSA > 340 mm^2^. A full stomach that increases risk of pulmonary aspiration of gastric contents in the event of general anesthesia was defined as Grade 2 regardless of CSA [2, 17].

For patients with full or intermediate stomach, consecutive ultrasound scan was performed every 10 min until empty stomach were detected. The antral cross-sectional area (CSA) was measured and recorded at each examination.

Patient characteristic data were recorded for analysis, including age, sex, Body Mass Index (BMI), ASA physical status classification, and scores of Self-Rating Anxiety Scale (SAS), fasting duration (defined as the time between the fluid or light meal ingestion and ultrasound examination), comorbidities, and surgery scheme. Other relevant data, including the postprandial plasma glucose concentration, hemoglobin A1c level and the diabetes complications, e.g., peripheral neuropathy defined as scores of Michigan Neuropathy Screening Instrument > 2 [19], cardiovascular autonomic neuropathy defined using the American Diabetes Association criteria and the Toronto Consensus Panel on Diabetic Neuropathy [20], diabetic nephropathy, and diabetes mellitus-related eye disease (including diabetic retinopathy, macular edema, rubeosis iridis, vitreous hemorrhage and diabetic related-visual injury) [21], were recorded for analysis, as well.

The primary outcome was the prevalence of full stomach in diabetic elective surgical patients. The secondary outcome was the gastric emptying time of clear liquids and light meal in diabetic patients. Using logistic regression analyses we examined the risk factors associated with full stomach.

### Sample size and statistical analysis

Based on the original data from our preliminary study and other study, the estimated occurrence of full stomach was 40% in diabetic patients, and 6.2% in elective surgical patients [22]. Thus, twenty-four patients per group would be expected to detect a significant difference with a type 1 error < 0.05 and a power of 80%. Taking into account a drop-out rate of about 10%, we originally plan to enroll 54 patients (27 in each group) to compare the incidence of full stomach. In order to investigate the risk factors for full stomach, we enlarged sample size to 108 patients for multivariate logistic regression analysis in our study (54 patients in each group). Statistical analysis was performed with SPSS 21.0(IBM Corp; Armonk, New York, USA).

After a Shapiro-Wilk test for normality of data distribution, continuous data (i.e., age, BMI, plasma glucose concentration, and hemoglobin A1c level) were expressed as the mean ± SD for normally distributed data, or median [interquartile range] for non-normally distributed data. The normally distributed continuous data were analyzed by student’s test and the non-normally distributed data were analyzed by Wilcoxon Rank Sum Test. Chi-square test or Fisher exact test were performed to compare incidence data (i.e., the percentage of co-morbidities, Perlas grade and the incidence of full stomach). Two-tailed tests will be used in all statistical analysis, and *P* value of less than 0.05 will be considered to be of statistical significance.

Univariate logistic regression analysis was used to identify variables associated with a full stomach, described as odds ratios (OR) with 95% confidence interval (*CI*). All variables that differed between groups (*P* < 0.05) together with the related-factors reported in previous studies were entered into a multivariate logistical regression analysis to investigate the risk factors for delayed gastric emptying in diabetic patients.

## Results

**Fig. 2:**
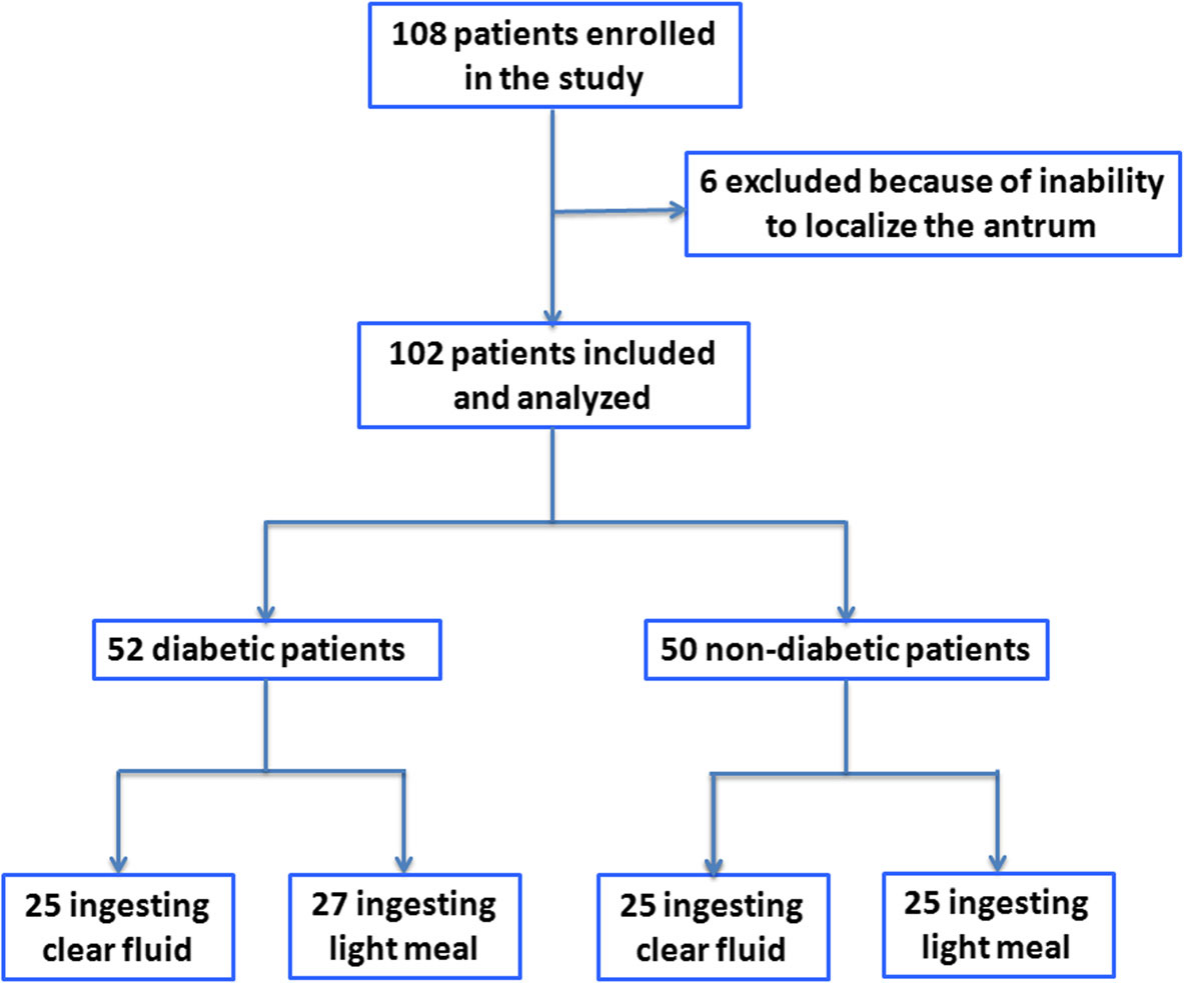
Flow chart and patients included in this study

One hundred and eight patients admitted for elective surgery were enrolled in this study: 54 type 2 diabetic patients and 54 non-diabetic controls. Two diabetic and four non-diabetic subjects were withdrawn from the study because of inability to localize the antrum. Finally, 102 patients (52 type 2 diabetic and 50 non-diabetic patients) completed the study and were included in the final analysis (Fig. 2), with the dropout rate at 5.56%.

There were no significant differences in age, sex, ASA physical status and BMI between diabetic and non-diabetic patients, except that the SAS scores were higher in diabetic patients, whereas both were lower than 40 (SAS score anxiety≥40 defined as anxiety) [23]. The main surgical plans for patients included were as follows: urological surgery (23.5%), gynecological surgery (27.5%), orthopedics surgery (11.8%) and others (37.2%), and no significant differences were observed between two groups (Table 1).

**Table 1:** Patients baseline characteristics

	Diabetic patient (*n* = 52)	Non-diabetic patient (*n* = 50)	*P*-value
Age (yr)	60.67 ± 10.34	60.25 ± 10.39	0.299
Body mass index (Kg.m^− 2^)	24.24 ± 2.83	22.34 ± 2.92	0.489
Sex (Female/male)	29/23	30/20	0.665
ASA physical status*			0.968
I	0 (0%)	12 (24.0%)	
II	50 (96.1%)	36 (72.0%)	
III	2 (3.9%)	2 (4.0%)	
Co-morbidities*			0.479
Cardiovascular disease	33 (63.5%)	32 (64.0%)	
Cerebrovascular disease	4 (7.7%)	6 (12.0%)	
Obesity#	5 (9.6%)	5 (10.0%)	
Scores of Self-Rating Anxiety Scale	28.75 ± 6.01	24.54 ± 4.12	0.001

Data are given as mean ± SD unless otherwise indicated

* Data are given as number (percentage of patients)

*ASA* = American Society of Anesthesiologists

# Defined by body mass index > 28

As shown in Table 2, type 2 diabetic patients have a higher prevalence of full stomach when compared to non-diabetic patients (48.1% vs. 8.00%, *P* = 0.000), which is 44.0% vs. 8.0% (*P =* 0.000) for 2-h fast after clear fluid and 51.9% vs. 8.0% (*P* = 0.000) for 6-h fast after a light meal, respectively.

**Table 2:** Quantitative and qualitative gastric ultrasound examination

	Diabetic patient	Non-diabetic patient	*P*-value
Clear liquid & light meal	n = 52	n = 50	
Perlas grade			0.000
0	6 (11.5%)	16 (32.0%)	
1	21 (40.4%)	30 (60.0%)	
2	25 (48.1%)	4 (8.0%)	
Antral cross-sectional area > 340 mm^2^ (Right lateral decubitus)	37 (71.2%)	25 (50.0%)	0.029
Indication			0.000
Empty stomach	13 (25.0%)	32 (64.0%)	
Intermediate stomach	14 (26.9%)	14 (28.0%)	
Full stomach	25 (48.1%)	4 (8.0%)	
Clear liquid	*n* = 25	n = 25	
Perlas grade			0.000
0	3 (12.0%)	10 (40.0%)	
1	11 (44.0%)	13 (52.0%)	
2	11 (44.0%)	2 (8.0%)	
Antral cross-sectional area > 340 mm2 (Right lateral decubitus)	18 (72.0%)	10 (40.0%)	0.023
Indication			0.000
Empty stomach	5 (20.0%)	15 (60.0%)	
Intermediate stomach	9 (36.0%)	8 (32.0%)	
Full stomach	11 (44.0%)	2 (8.0%)	
Light meal	*n* = 27	n = 25	
Perlas grade			0.000
0	3 (11.1%)	6 (24.0%)	
1	10 (37.0%)	17 (68.0%)	
2	14 (51.9%)	2 (8.0%)	
Antral cross-sectional area > 340 mm2 (Right lateral decubitus)	19 (70.4%)	15 (60.0%)	0.432
Indication			0.000
Empty stomach	6 (22.2%)	17 (68.0%)	
Intermediate stomach	7 (25.9%)	6 (24.0%)	
Full stomach	14 (51.9%)	2 (8.0%)	

Data are given as number (percentage of patients)

For patients with full or intermediate stomach following fasting guidelines (39 diabetic and 18 non-diabetic), consecutive ultrasound examination was performed every 10 min until empty stomach was detected. The average time to empty stomach was significantly longer in type 2 diabetic patients than that of non-diabetic patients, being 146.50 ± 40.91 mins vs. 124.50 ± 10.68 mins (*P* = 0.014) after ingesting clear liquids and 426.50 ± 45.25 mins vs. 370.00 ± 53.97mins (*P* = 0.042) after light meal, respectively.

In order to investigate the risk factors for full stomach in diabetic patients, we further divided diabetic patients into full stomach group and empty/intermediate stomach group based on gastric ultrasound grade and performed a subgroup analysis. The baseline characteristics (age, sex, BMI and scores of self-rating anxiety scale) showed no significant difference between subgroups. The median duration of diabetes were 6 years (IQR [2–10 yr]), and the treatment medicine for diabetes showed no significant difference between subgroups. Interestingly, the prevalence of diabetes mellitus-related eye disease was higher in patients with full stomach after recommended fasting duration, than those with empty or intermediate stomach (52.0% vs. 22.2%, *P* = 0.026). However, the incidence of peripheral neuropathy and cardiovascular automonic neuropathy showed no significant difference between groups, and none of the patients had diabetic nephropathy (Table 3). Further univariate analysis showed that diabetes-related eye disease was significantly associated with full stomach (OR = 4.83, *P* = 0.010), while we did not find any association of age, Hemoglobin A1c, peripheral neuropathy, and cardiovascular autonomic neuropathy with full stomach. After adjusted by age, sex, BMI and SAS scores, diabetes-related eye disease was shown to be an independent risk factor of full stomach (Table 4).

**Table 3:** Diabetic patients’ characteristics between those with full stomach and empty/intermediate stomach

Characteristic	Full stomach (n = 25)	Empty/intermediate stomach (n = 27)	*P*-value
Age (yr)	62.21 ± 10.27	58.83 ± 10.88	0.553
Body mass index (Kg.m^−2^)	24.34 ± 3.19	23.79 ± 2.93	0.563
Sex (Female/male)	13/12	14/13	0.991
Scores of Self-Rating Anxiety Scale	28.74 ± 5.82	28.26 ± 6.25	0.800
Fasting glucose (mmol/L)	7.88 ± 2.79	7.80 ± 2.40	0.921
Postprandial glucose (mmol/L)	10.50 ± 4.05	10.69 ± 2.48	0.861
Hemoglobin A1c (%)	7.5 ± 1.7	7.3 ± 1.6	0.861
Diabetic nephropathy	0 (0%)	0 (0%)	–
Peripheral neuropathy*	6 (24.0%)	6 (22.2%)	0.879
Cardiovascular autonomic neuropathy *	18 (72.0%)	18 (66.7%)	0.677
Diabetes mellitus-related eye disease*	13 (52.0%)	6 (22.2%)	0.026

Data are given as mean ± SD unless otherwise indicated

*Data are given as number (percentage of patients)

**Table 4:** Predictors of full stomach in diabetic patients

Parameter	Univariate analysis	Multivariate analysis		
	Odds ratio (95% CI)	*P*-value	Odds ratio (95% CI)	*P*-value
Age	0.459 (0.957, 1.066)	0.647		
Sex	0.813 (0.337, 3.123)	0.578		
Body mass index	0.987 (0.811, 1.202)	0.900		
Scores of Self-Rating Anxiety Scale	1.000 (0.911, 1.097)	0.995		
Hemoglobin A1c	1.066 (0.738, 1.540)	0.733		
Peripheral neuropathy	0.773 (0.211, 2.826)	0.697		
Cardiovascular autonomic neuropathy	0.792 (0.128,5.561)	0.447		
Diabetes mellitus-related eye disease	4.825 (1.467,15.873)	0.010	4.825 (1.467,15.873)	0.010*

Data are expressed as odd ratio (95% *CI*)

*Adjusted by Age, Sex, Body mass index and Scores of Self-Rating Anxiety Scale

## Discussion

This prospective study showed that almost half of the type 2 diabetic patients with a median duration of 6 years had a full stomach following the current preoperative fasting guideline, and the average time to empty stomach state for diabetic patients is 146.50 ± 40.91 mins for clear liquids and 426.50 ± 45.25 mins for light meal, longer than the recommended fasting duration of ASA [5]. Furthermore, we found patients with diabetes mellitus-related eye disease are at significantly increased risk of full stomach compared to those without (OR = 4.83, *P* = 0.010).

The diabetes population is important to study for several reasons. First, diabetes mellitus currently affects 10–15% of surgical patients worldwide, and this number is further increasing dramatically [24]. It is estimated that more than 382 million people have diabetes mellitus nowadays, and the number affected will reach 592 million by year 2035 [24]. Second, delayed gastric emptying occurred in almost half of longstanding diabetic patients. Thus, diabetic patients should be considered at high risk of pulmonary aspiration during the perioperative period, which are still a contributing cause of death perioperativel y[18]. Therefore, a noninvasive and more easily available technique to determine whether full stomach exists, for anesthesiologists to individualize assessment of the risk of pulmonary aspiration and finally to enhance perioperative safety, is in urgent needed.

Ultrasound has been proposed as a point-of-care test to assess gastric volume and the risk of pulmonary aspiration, and anesthesiologists might become proficient in gastric ultrasound assessment after a short training session [25]. In the present study, full and empty stomach were defined using Perlas qualitative grading scale, combining with the measurement of the antral cross-sectional area in the RLD position [17]. We found that 48.1% of the type 2 diabetic patients had a full stomach according to the current preoperative fasting guideline, suggesting the high risk of regurgitation and pulmonary aspiration in the event of general anaesthesia. The findings determined by antrum ultrasound examination were in accordance with previous studies [26–28]. Thus, when general anesthesia is required for a patient with full stomach, rapid sequence induction and tracheal intubation are indicated. Furthermore, following a consecutive ultrasound scan, we detected that the average time of empty stomach in diabetic patients is longer than the fasting time recommended by ASA, which indicated that the fasting duration should be prolonged for certain diabetic patients.

The prevalence of delayed stomach emptying in diabetic patients was reported to be associated with autonomic neuropathy, retinopathy, and nephropathy [29]. Consistently, in the present study, the incidence of diabetes mellitus-related eye disease is 36.5% (19/52), similar to Burgress’s reports [30], and univariate analysis demonstrated that diabetes mellitus-related eye disease was significantly correlated with delayed stomach emptying, with up to a fivefold increased risk of full stomach compared to those without diabetes mellitus-related eyes disease. Previously study showed that autonomic neuropathy and enteric neuropathy plays an important role in the pathogenesis of diabetic gastroparesis [31]. Coincidently, more recent findings suggest that neurodegeneration also plays a critical role in the pathogenesis of diabetic retinopathy [32, 33]. Thus, we hypothesized that neuropathy, as the same underlying mechanism for both gastroparesis and retinopathy, might partly explain why diabetes mellitus-related eye disease was significantly correlated with delayed stomach empty in diabetic patients. Although, an in-depth discussion of the relationship with eye disease and delayed gastric emptying is far beyond the purpose of this study, it first highlighted that preoperative fasting time might need to be longer for diabetic patients with related eye disease. Further studies are therefore warranted to validate our hypothesis.

Surprisingly, we did not detect significant correlation between BMI with delayed stomach emptying, inconsistent with previous studies, which showed obesity was a risk factor for delayed stomach emptying [34]. This is possibly due to the fact that a relatively small sample size of obese patients was recruited. Another reason is that our study limited patients with a body mass index (BMI) < 35 kg/m^2^. Of note, previous studies reported divergent findings regarding the impact of serum glucose concentration, hemoglobin A1c concentration and “early” type 2 diabetes on stomach emptying. Some have proposed that gastric emptying is often accelerated in patients with “early” Type 2 diabetes [35]. However, some have claimed that high glycaemia and hemoglobin A1c concentration were correlated with gastric emptying time [36]. Nevertheless, others showed it was the acute changes in the blood glucose concentration, not glucose concentration that affects gastric emptying [37, 38]. In the present study, we did not find any relationship between serum glucose or hemoglobin A1c concentration with delayed stomach emptying. Moreover, in our study, none of the patients had diabetic nephropathy. That’s may partly because the duration of diabetes in our study is not long enough, with only 6 years (IQR 2–10 years). Therefore, further studies with a larger sample size are needed to clarify the issues.

This study has several limitations. Firstly, all diabetic subjects enrolled for this study were with type 2 diabetes, and only a minority of diabetic patients with complications, which might be insufficient to determine other predictive factors of delayed stomach emptying. Therefore, our results may be only applicable to type 2 diabetic patients with similar characteristics. Secondly, ultrasound examination was performed after patients’ admission to the surgical department, while not in the immediate preoperative period before anesthesia, because predicting the timing of an operation is often inaccurate and the surgical schedule is frequently subject to changes. Thus, our findings might possibly not represent the condition before anesthetic induction. However, in clinical practice, we suggest that preoperative ultrasound assessment of gastric content should be performed for patients with diabetes duration of 6 years or more. Moreover, a prokinetic drug and rapid sequence induction is recommended for such cases.

## Conclusions

Ultrasound examination could be used as a point-of-care test to predict gastric contents in patients with type 2 diabetes-related eye disease and our results showed that 48.1% of diabetic patients had a full stomach following the current preoperative fasting guidelines in this cohort. Patients with diabetic-related eye disease are at significantly increased risk of delayed gastric emptying. Other studies may be needed to further investigate the relationship of stomach emptying and mild, moderate, and severe diabetic patients and those with complications. To conclude, we suggest that preoperative ultrasound assessment of gastric content should be performed in all type 2 diabetic patients, especially those with diabetes mellitus-related eye disease.

## Data Availability

The datasets analyzed during the current study are available from the corresponding author on reasonable request.

## Abbreviations

ASA: American Society of Anesthesiologists
BMI: Body Mass Index
CSA: Cross-sectional area
RLD: Right lateral decubitus
SAS: Self-Rating Anxiety Scale
